# Mobile Laser Scanning in Forest Inventories: Testing the Impact of Point Cloud Density on Tree Parameter Estimation

**DOI:** 10.3390/s25185798

**Published:** 2025-09-17

**Authors:** Nadeem Ali Khan, Giovanni Carabin, Fabrizio Mazzetto

**Affiliations:** Faculty of Agricultural, Environmental and Food Sciences, Free University of Bozen-Bolzano, Piazza Università 1, 39100 Bolzano, Italy; nadeemali.khan@unibz.it (N.A.K.); fabrizio.mazzetto@unibz.it (F.M.)

**Keywords:** LiDAR, point cloud density, robotic platform, forest inventories

## Abstract

Forest inventories are essential for monitoring and managing forest ecosystems, relying on accurate measurements of tree attributes such as tree detection, Diameter at Breast Height (DBH), and Tree Height (TH). Nowadays, advances in LiDAR technology have enabled increasingly effective and reliable solutions for 3D mapping and tree feature extraction. However, the performance of this method is strongly influenced by point cloud density, which can be limited for technological and/or economic reasons. This study therefore aims to investigate and quantify the effect of density on the accuracy of measured parameters. Starting from high-density datasets, these are progressively downsampled, and the extracted features are compared. Results indicate that DBH estimation requires densities of 600–700 points/m^3^ for errors below 1 cm (5% RMSE), while accurate tree height estimation (RMSE < 1 m—5% error) can be achieved with densities exceeding 300 points/m^3^. These findings provide guidance for balancing measurement accuracy and operational efficiency in automated forest surveys using laser scanner technology.

## 1. Introduction

Forest inventories play a vital role in accurately estimating tree parameters (e.g., tree identification, Diameter at Breast Height, Tree Height), which are crucial for effective forest monitoring, management, mapping, scientific research, and future planning [1,2,3]. Decades ago, forest data collection relied on manual methods. While widely used, these approaches were time-consuming, laborious, costly, and prone to operator errors during manual calculations and recording [4,5,6,7,8]. In recent years, advances in digital technologies have greatly improved the efficiency and accuracy of extracting tree-level features across entire forest plots, particularly through LiDAR-based methods [9,10]. In the specific domain of forest monitoring, LiDAR is primarily employed through two principal approaches, airborne surveys and under-canopy scanning, each targeting different spatial scales and levels of detail.

In Airborne Laser Scanning (ALS) [11,12], performed from aircraft or Unmanned Aerial Vehicles (UAVs), measurements mainly capture the forest canopy from above. In this approach, canopy structure is directly measured, tree height can sometimes be estimated, and other forest attributes are typically derived through statistical modeling. Its main drawback is reduced accuracy in detecting sub-canopy tree attributes due to beam broadening at long ranges, canopy occlusion, and partial trunk coverage [13].

The second approach focuses on under-canopy measurements. Terrestrial Laser Scanning (TLS), based on fixed ground-based positions, delivers in an effective way highly accurate and detailed data, particularly for trunk-level features [14,15,16,17]. Through various techniques, this fixed scanning position can be moved, and the resulting scans can be merged into a comprehensive point cloud. TLS is particularly suited to small areas, providing high-resolution scans within a range of just a few tens of meters. Although it is possible theoretically to apply TLS across large forested areas, the process is time-consuming and typically requires hundreds of individual scans to achieve full coverage [2]. A more advanced alternative is Mobile Laser Scanning (MLS) [18,19], more broadly known as mobile mapping systems, which relies on moving platforms such as handheld devices, backpack, or robotic units to capture under-canopy data. The system has also proven suitable for large-scale forest inventories that include under-canopy measurements [20]. However, in complex forest environments, where GNSS signals are often obstructed by dense canopy cover, the Simultaneous Localization and Mapping (SLAM) algorithm is employed to improve positioning accuracy [21,22]. MLS is well suited for large forest plot surveys, as it offers significant time saving and improved under-canopy data capture. Compared with TLS, it allows for a reduction in occluded areas, which enhances overall data completeness [23].

This approach is adopted within the project “An Artificial Intelligence approach for FOrestry Robotics in Environment Survey and inspecTion” (AI4FOREST), to which the present work is related. The project focuses on developing a ground-based robotic platform for forest inventory, based on AgileX Scout 2.0 rover, capable of autonomously navigating complex forest environments. The system determines optimal paths while accounting for terrain conditions and reconstructs the 3D environment using SLAM [24]. The resulting point cloud is then used to extract key forest attributes, including individual tree detection, positional mapping, Diameter at Breast Height (DBH), and Tree Height (TH).

Once LiDAR data has been acquired through either airborne, terrestrial, or mobile scanning approaches, the next essential step is to process the resulting point clouds to extract meaningful forest information. A variety of commercial and open-source tools have been developed to support these tasks, offering visualization, analysis, and point cloud processing capabilities. Some of these have been specifically adapted for forestry applications. Examples include LiDAR360^®^ [25], ForestView^®^ [5,26], TerraScan [27,28], Leica Cyclone [29,30], and CompuTree [31,32]. Among open-source solutions, CloudCompare is widely used [3,33,34]. Additionally, the literature reports the development of several specialized tools and libraries designed specifically for forest inventory applications. These tools typically process raw point cloud data to identify individual trees and extract various tree-level attributes. Examples include TreeSeg [4] and 3DForest [2,35,36]. Moreover, several relevant libraries are available in Python, with a few integrated into R packages.

While numerous tools and libraries exist to extract forest attributes from LiDAR point clouds, the accuracy of these analyses critically depends on the quality and density of the acquired data.

As previously mentioned, one of the main challenges with laser scanning systems is that achieving precise measurements of forest structural parameters requires highly dense point clouds. However, increasing point cloud density typically requires more advanced and costly acquisition systems capable of higher sampling rates, or alternatively, slowing down the rover speed—both of which increase survey time and demand more precise positioning. To address this trade-off, this study specifically investigates the impact of point cloud density on the accuracy of extracting key forest inventory metrics.

The primary objective is therefore to determine the optimal and minimum point cloud density from Mobile Laser Scanning (MLS) data that balances measurement accuracy with operational efficiency in forest surveys. Starting from several high-density datasets, key forest inventory features (i.e., DBH and TH) have been extracted using the 3DFin tool library [1,37]. The dataset density has been then progressively and artificially reduced to assess the impact on result accuracy. Previous research has investigated optimal density requirements for Terrestrial Laser Scanning (TLS) with both single and multi-scan approaches [38,39]. However, to date, no study has systematically examined this aspect for MLS systems. Unlike TLS, MLS continuously acquires point clouds along a trajectory, and its performance is affected not only by point density but also by factors such as carrier speed, terrain complexity, understory vegetation, and the proximity of neighboring stems [21]. Establishing density thresholds for MLS is therefore essential for operational forest inventories.

## 2. Materials and Methods

This section presents the methodology used to determine the optimal point density for Mobile Laser Scanning (MLS) acquisitions, ensuring sufficiently accurate recognition of key forest features. This study is based on the analysis of high-density forest datasets freely available online. First, the MLS-derived datasets employed in this work are introduced and described. The methodology and algorithms applied to progressively reduce point cloud density across these datasets are then outlined. Subsequently, the tools and tasks used to extract tree features—specifically Diameter at Breast Height (DBH) and Tree Height (TH)—from the progressively downsampled datasets are presented. Finally, the methodology adopted to compare and evaluate the obtained results is explained.

### 2.1. Forest Datasets

**Figure 1:**
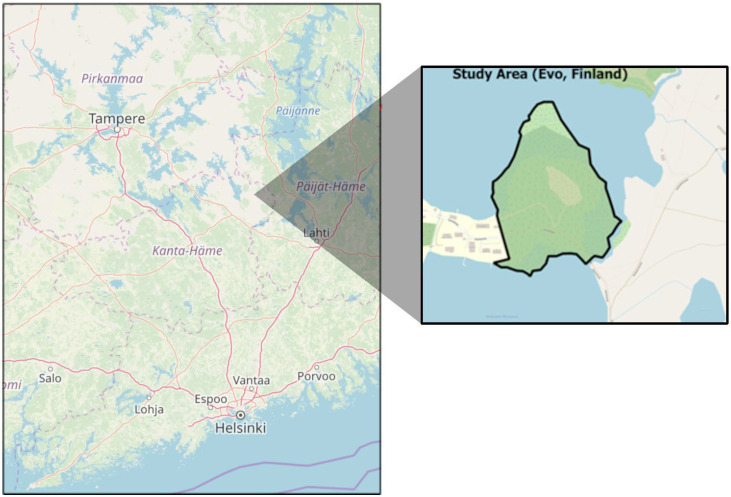
Location of the forest plots in Evo region, Finland.

Two different open-source datasets were considered in this study. The first dataset [19,40], hereafter referred to as Forest 1, was collected in southern Finland, within the Evo region (61.19° N, 25.11° E). A map showing the location of the study forest is provided in Figure 1. This experimental site consists of a naturally managed boreal forest characterized by a mixed-species stands, dominated by Scots pine (*Pinus sylvestris* L.), Norway spruce (*Picea abies* (L.) H. Karst.), broadleaved silver birch (*Betula pendula* Roth), downy birch (*Betula pubescens* Ehrh.), and European aspen (*Populus tremula* L.). The second dataset [1,41], referred to as Forest 2, is openly accessible via the 3DFin platform. It includes approximately 43.76 million points covering a total area of 684 m^2^. Unfortunately, there is no reference regarding the forest location and characteristics. Both datasets were acquired using the Zeb-Horizon–GeoSLAM Ltd., Nottingham, United Kingdom–Mobile Laser Scanner.

To reduce and limit the computational demand in terms of RAM, CPU, and GPU resources, each dataset was divided into three smaller sub-units, and they all were processed individually. These subsets were renamed as plots A, B, and C for forest 1, while plots D, E, and F refer to forest 2.

### 2.2. Reduction of MLS Point Cloud Density

**Figure 2:**
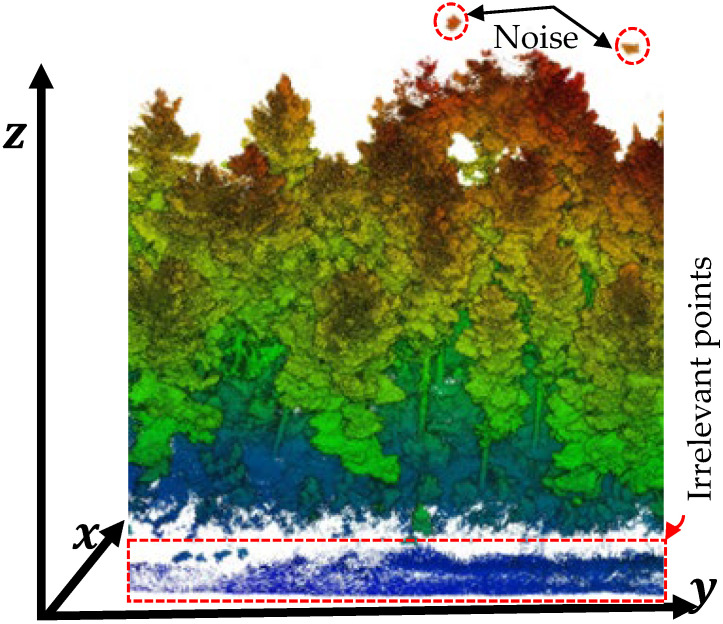
Visual inspection of unnecessary points and noise before filtering.

Mobile Laser Scanning (MLS) systems capture dense 3D point clouds that include not only forest structures but also additional elements such as ground vegetation, stones, roads, buildings, and moving objects (e.g., vehicles, pedestrians). Non-forest objects were removed from the dataset through manual segmentation in CloudCompare to retain only forest-related points (Figure 2). After visual inspection confirmed that only relevant points remained, the data was used to estimate point density (i.e., number of points per cubic meter—points/m^3^). The spatial extent of the dataset was defined using the dimensions of an axis-aligned bounding box (ΔX, ΔY, ΔZ), calculated from the minimum and maximum coordinates.

This density value serves as a metric for quantifying the concentration of points in 3D space. It is particularly sensitive to variations in the vertical structure of the canopy, as it reflects both the horizontal distribution and the vertical layering of vegetation elements, such as leaves and branches, within the defined volume. Table 1 presents the calculated density values for each subplot across the two forest sites.

**Figure 3:**
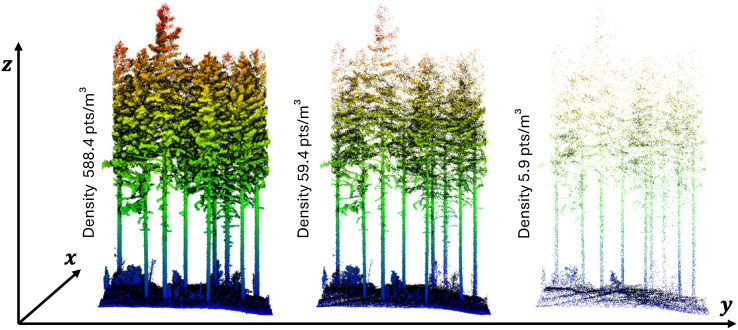
Comparison between different levels of point cloud density: **left**: 100%, **center**: 5%, and **right**: 1% of the total density.

Starting from the initial full density point cloud, the various subplots were progressively thinned by gradually reducing point density, as illustrated in Figure 3. The original point cloud (100% of points) served as the reference for all reductions. Subsets were generated by sequentially retaining 90% to 10% of the original points in 10% increments, followed by additional subsets ranging from 9% to 1% in 1% increments. Crucially, each reduced subset was derived directly from the original full-density point cloud, rather than from already thinned versions. This approach ensured that each subset maintained a consistent and unbiased random distribution of points. Point cloud sampling was performed in CloudCompare software (v2.13.2, Palaiseau, France) using the random sampling by percentage tool to reduce point densities consistently across all three plots in both forest 1 and forest 2. In total, this procedure produced 19 distinct point cloud versions (ranging from 100% to 1% density) for each of the forest areas studied.

### 2.3. Tree Features Recognition Process

The analysis of the point cloud and the identification of the different forest features were carried out using the 3DFin library and CloudCompare software. The point cloud was processed following a structured workflow with three main steps, detailed below: (1) trees height normalization (2) identification of individual trees, (3) measurement of tree features (i.e., DBH and TH).

The first step was the normalization of tree heights, achieved by subtracting the ground profile (Digital Terrain Model—DTM) from off-ground points. This ensured that the base of all trees was adjusted to the same reference height. In other words, the DTM served as the reference surface for estimating key tree attributes, including Tree Height, Diameter at Breast Height (DBH), and stem curve. The accuracy of these derived metrics is highly dependent on DTM precision, as a more accurate terrain model improves the reliability of individual tree parameter estimations [2]. The DTM was generated using the Cloth Simulation Filter (CSF) algorithm, a built-in function in CloudCompare software, as shown in Figure 2. The CSF algorithm is particularly effective for forested environments, as it filters out non-ground elements such as shrubs and stones, even under complex topographic conditions such as uneven or sloped terrain [42]. In particular, a DTM resolution of 0.45 m was selected to balance detail and computational efficiency.

For trees identification, the primary step was to identify the tree stems. To do this, a stripe was extracted from the normalized point cloud. The lower height of the stem was set at 0.3 m from the ground surface while the upper height was considered 5 m. The points within this stripe were then voxelized, and their verticality was computed as suggested by [43].

Tree Height was calculated following the methodology proposed by [11]. The point cloud was first clustered and voxelized using the DBSCAN algorithm. Large clusters corresponding to trees were retained, while smaller clusters were discarded. Tree Height was then derived from the distribution of voxels along the z-axis.

**Figure 4:**
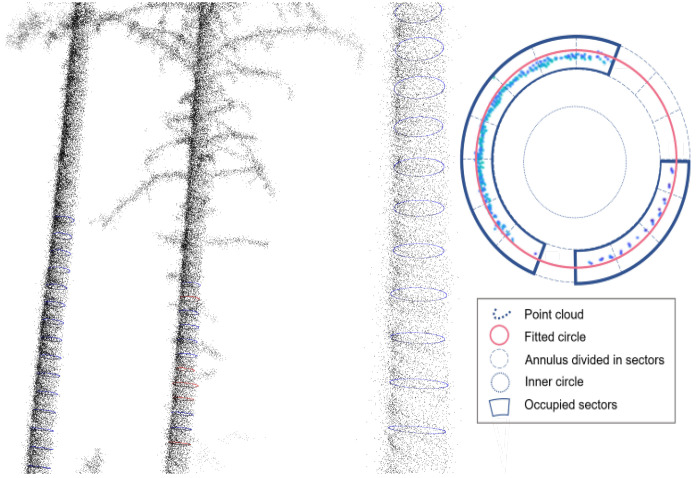
Circle fitting for the estimation of DBH.

The final step, following the successful extraction of tree stems, was the calculation of DBH. A 0.02 m slice of each stem was taken at a height of 1.3 m (i.e., breast height). Circle fitting (Figure 4) was then performed on this slice using the least squares method as proposed by [11]. An outer circle was fitted around the points representing the stem surface, and a smaller inner circle, with the same center but a smaller radius (e.g., half the size), was placed inside. Most points should lie outside the inner circle, representing the stem surface; if too many points fall inside, the outer circle may be incorrectly fitted. Additionally, the area around the circle was divided into small sectors to ensure that each sector contained points. If too many sectors were empty, the points might not properly represent the stem, making the circle fitting unreliable.

### 2.4. Performance Metrics

To comprehensively evaluate the performance of forest tree attribute detection, standard metrics and benchmarks are typically employed. One such metric is completeness, which estimates the proportion of correctly detected trees relative to a reference dataset. It is calculated as:

(1) $$completeness=\frac{number\;of\;detected\;trees}{number\;of\;actual\;ground\;truth\;trees}\times 100$$

Another key metric is the Root Mean Square Error (RMSE), which quantifies the average deviation between detected and reference values for tree attributes such as DBH and TH. RMSE is computed by taking the squared differences between the detected and reference values, averaging them, and then taking the square root. This provides an indication of the magnitude of error present in the estimates.

In addition, bias quantifies systematic error between the observed and actual values. Bias can be positive (overestimation), negative (underestimation), or zero (no systematic error). For example, a positive bias in DBH indicates that the LiDAR-derived value exceeds the actual DBH, while a negative bias suggests underestimation. A zero bias implies that the predicted and actual values are identical.

In the absence of field-verified ground truth data, the high-density (100%) point cloud was used as a reference dataset. Due to the detailed canopy structure and favorable visibility conditions at this density, detection accuracy is assumed to be high. This approach, similar to that adopted in [33], allows for the computation of relative completeness, RMSE, and bias across lower-density scenarios. While not a true ground truth, this high-density dataset serves as a consistent and reliable benchmark for comparative evaluation.

## 3. Results

Table 2 summarizes the structural characteristics of the six sample plots from the two boreal forests. All plots have the same surface area (228 m^2^), but point cloud density varies considerably, ranging from 588 points/m^3^ in plot D (Forest 2) to 3677 points/m^3^ in plot F (forest 2). Forest 1 is characterized by shorter trees (average height 12–13 m) and more uniform stem Diameters at Breast Height (DBH, 0.18–0.21 m). In contrast, forest 2 contains taller trees (average height 18–22 m) and shows greater variability. This is most evident in plot D, where the maximum DBH reaches 0.49 m due to low vegetative cover, which created more growing space for individual trees to expand in both height and diameter. Overall, forest 1 displays a relatively uniform canopy, while forest 2 exhibits higher heterogeneity in both tree size and spatial distribution.

**Figure 5:**
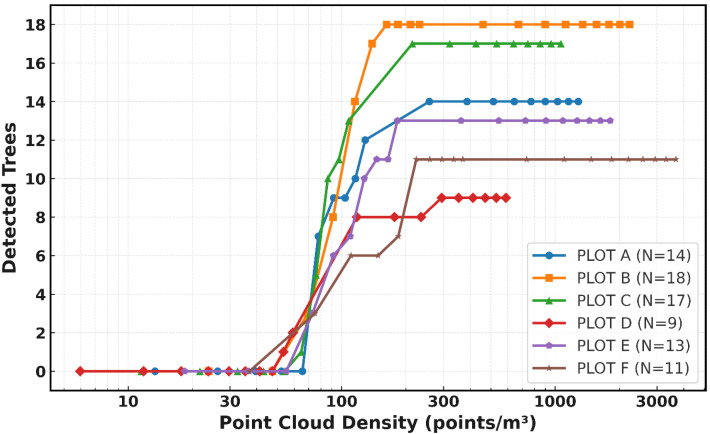
Tree detection rate as a function of point cloud density across forest plots.

**Figure 6:**
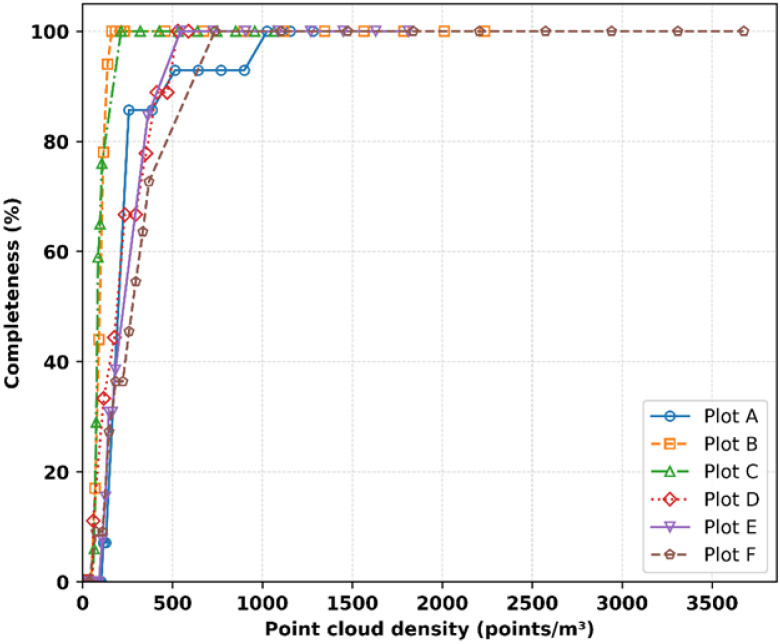
Relationship between point cloud density and completeness for plots A–F.

Tree detection and completeness increase consistently with point cloud density (Figure 5 and Figure 6). At densities above approximately 100 points/m^3^, most plots achieve 80–90% detection, indicating a sharp rise. Bienert et al. [44] reported 96% of detection using a distance-adaptive segmentation approach, while Tupinambá-Simões et al. [45] reported 88–92% using ALS. As density increased further, detection approached ~100% in all plots once densities exceeded ≈ 200 points/m^3^. Completeness followed a similar trend, showing greater variability below ~160 points/m^3^ but stabilizing near 100% between 160 and 300 points/m^3^. These thresholds are comparable to those reported for MLS and TLS in other boreal and temperate forests [3].

**Figure 7:**
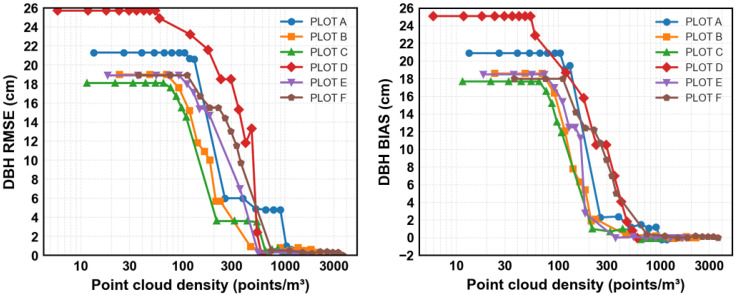
DBH estimation accuracy and bias across forest plots at varying point cloud densities.

Figure 7 shows DBH measurement benchmarks, with RMSE on the left and bias on the right. At low point cloud densities (<200 points/m^3^), RMSE exceeds 20 cm due to incomplete stem profiles and noise affecting circle fitting. As density increases, errors decrease rapidly: RMSE falls below 2 cm (~10% of average DBH) at ~600–700 points/m^3^ and stabilizes under 1 cm at ≥1000 points/m^3^. This threshold is slightly lower than that reported by [23], who achieved 1.11 cm RMSE using multi-scan MLS. This improvement likely reflects the simpler vertical structure and good visibility of the boreal plots considered in this work, as well as smaller sub-units reducing occlusion. Our results also compare favorably with TLS-based methods; for example, Krasinski et al. [20] reported 10.3 cm RMSE when combining TLS with the Forest Structural Complexity Tool, notably higher than our MLS results at comparable densities.

Bias patterns closely follow RMSE trends, with positive values indicating slight DBH overestimation. Above 600 points/m^3^, bias remains below 0.5 cm, comparable to the 0.08 cm reported by [23]. The overestimation likely results from residual bark roughness and partial occlusions that shift circle fits outward.

**Figure 8:**
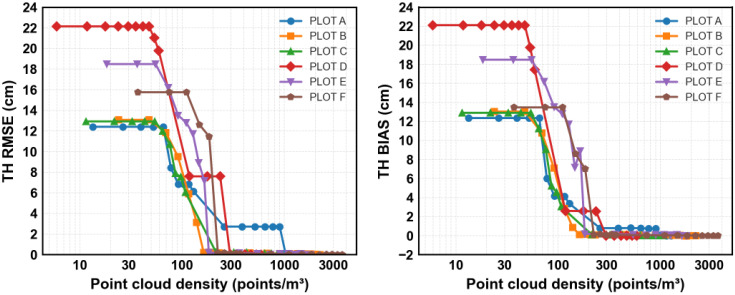
Tree height estimation accuracy and bias across forest plots at varying point cloud densities.

Figure 8 shows benchmarks for Tree Height (TH) estimation. RMSE decreases from 2.73 m at low densities to 0.18 m at the highest tested. Errors decrease sharply up to ~300 points/m^3^ then stabilize below 1 m (≈5% of average tree height, 15–20 m). This threshold is consistent with [3], who reported minimal accuracy loss at densities down to ~136 points/m^2^, although in boreal conditions our results indicate further improvement up to ~300 points/m^3^. Bias remains low across densities above 300 points/m^3^, with values typically within ±0.2 m. The slight positive bias observed in some plots suggests a tendency to slightly overestimate Tree Heights, possibly due to misclassification of outlier points (e.g., small branches or noise above the true apex) as part of the treetop.

## 4. Discussion

### 4.1. Discussion on Methodological Limitations

The main limitation of this study includes the reliance on open-source MLS datasets that have no availability of ground-truth data (i.e., tree properties collected directly in the field using conventional measurements). Indeed, in this work, 100% density has been considered as the reference data for the comparison. This implies that the retrieved results are not absolute but relative to the higher-density map. In any case, this is important, as it provides a quantification of the loss of resolution compared to very detailed surveys acquired with the state-of-the-art technology. Similar approaches can be found in the literature, such as in [33], where the influence of scan density (evaluated qualitatively) and acquisition path using Hand-Held Mobile Laser Scanning was investigated by comparison with detailed acquisitions around individual stems.

Moreover, this work was limited to tree detection, DBH, and TH. Other forest attributes such as biomass, canopy diameter, and stem volume were not considered, as they may require different density thresholds. However, the selected parameters represent the most commonly measured and required forest variables.

### 4.2. Discussion of the Results

The error patterns observed in the two forests can be explained by differences in canopy structure, under-story vegetation, and terrain. In forest 1, the terrain was slopy, producing shadow and occlusion effects, but DBH and TH remained relatively stable until densities dropped below ~20% of the original. This suggests that once the stem cross-section has sufficient clusters, the accuracy of DBH estimation is maintained. Similar findings have been reported in TLS and MLS studies, which showed that the completeness of stem coverage is more important than the total number of points recorded [33,38]. Forest 2 showed modest variability due to flat terrain, heterogeneity, and canopy gaps, leading to consistent accuracy across all plots except plot D due to low vegetation and fewer clusters resulting in low density, exhibited high error, while plots E and F retained high accuracy.

The tree detection rates observed in this study are in line with previous work using mobile and personal laser scanning. Bauwens et al. (2016) [23] segmented 91 percent of trees in mixed natural forests with handheld MLS. Zhao et al. (2018) [46] and Wu et al. (2013) [47] reported over 92% and 98% completeness for street tree detection, respectively. Gollob et al. (2020) [48] demonstrated detection rates exceeding 96% using a personal laser scanner, while Hyyppä et al. (2020) [21] achieved stem detection completeness between 88% and 95% with density-based filtering criteria.

For DBH estimation, MLS and personal laser scanning studies have reported RMSE values ranging from 1.58 to 4.78 cm across different forest types [33,48,49]. The RMSE values observed in this study, ranging between 1.97 and 3.31 cm, fall within this interval. This confirms that MLS-derived DBH estimates are comparable in accuracy to other state-of-the-art approaches. Tree Height estimation was also in line with previous findings that highlighted the sensitivity of height metrics to point density and canopy occlusion [50,51]. These results indicate that beyond point density, structural complexity, understory vegetation, and slope-induced occlusion also influence error margins. Similar patterns have been reported in TLS and airborne laser scanning (ALS) studies, where increasing point density beyond a minimum threshold did not always improve the prediction of forest parameters [3,39].

### 4.3. Implications of the Results

The results show that the accuracy of MLS-derived forest parameters depends not only on point density but also on canopy structure and occlusion. DBH requires higher point density because incomplete coverage of the stem cross-section leads to high errors, whereas Tree Height can be estimated once the upper canopy is well captured, and further increases in density provide greater accuracy as compared to DBH. Apart from density, several other factors like area and geometry of the forest, stand conditions, and processing approach can influence the estimation of the forest metrics. The density ranges observed here are useful for initial planning of MLS surveys, as they suggest how much data is needed to achieve reliable accuracy while keeping acquisition time and data volume manageable. This is particularly relevant for autonomous robotic platforms being developed in the AI4FOREST project. Future studies should include field measurements to establish absolute accuracy and extend the analysis to other attributes such as crown size, stem diameter, and biomass. Combining MLS with other sensing platforms, including TLS and UAV LiDAR, will also help to evaluate whether these density requirements can be applied across different forest types and acquisition systems.

## 5. Conclusions

Advances in Mobile Laser Scanning have greatly improved the ability to acquire three-dimensional information on forest structure, including detailed under-canopy measurements. The reliability of tree detection and parameter estimation is strongly influenced by point cloud density. This study assessed the minimum and optimal densities required for accurate estimation of Diameter at Breast Height (DBH) and Tree Height (TH) using two open-source MLS datasets. Point cloud densities were systematically reduced and evaluated against reference measurements from full-density datasets.

The results indicate that DBH estimation requires relatively high densities, with good results achieved at approximately 600 to 700 points/m^3^, yielding RMSE errors of less than 1 cm. In comparison, TH estimation was acquired with accurate results obtained at densities exceeding 300 points/m^3^ and RMSE errors below 1 m. Similar patterns were observed for bias in both DBH and TH. These findings confirm that MLS can support accurate forest inventories at reduced point densities, but they also highlight the need to maintain sufficient sampling levels to avoid error inflation, particularly for DBH.

Future work should extend these analyses to additional forest attributes such as canopy volume and biomass and should incorporate MLS data collected by autonomous robotic platforms in combination with comprehensive ground-truth measurements. This will further refine density thresholds and improve the operational application of MLS in forest resource monitoring.

## Figures and Tables

**Table 1:** Point cloud densities (points/m^3^) for each plot in both forests at full data resolution (100% density).

Forest	Plot	Density (Points/m^3^)
1	A	2234.5
	B	1282.2
	C	1064.3
2	D	588.44
	E	1810.84
	F	3677.03

**Table 2:** Summary of forest plots and their associated attributes.

		Forest 1			Forest 2	
Plot Characteristics	Plot A	Plot B	Plot C	Plot D	Plot E	Plot F
Surface area (m^2^)	228	228	228	228	228	228
Point cloud (M Pts)	15.97	8.94	7.55	3.35	15.97	16.40
Density (points/m^3^)	2234	1282	1064	588	1810	3677
Number of trees	18	14	17	9	13	11
DBH mean (m)	0.19	0.21	0.18	0.23	0.19	0.20
DBH max (m)	0.25	0.27	0.24	0.49	0.25	0.22
DBH min (m)	0.13	0.12	0.10	0.17	0.11	0.17
TH mean (m)	13	12.40	12.90	22.03	18.31	18.44
TH max (m)	15.15	14.06	14.77	24.53	20.38	20.42
TH min (m)	11.23	10.22	10.58	20.46	14.88	17.05

## Data Availability

The original contributions presented in this study are included in the article. Further inquiries can be directed to the corresponding author(s).
